# Effect of Mg Addition on Inclusions in the Welding Heat-Affected Zone of Pressure Vessel Steels

**DOI:** 10.3390/ma16237369

**Published:** 2023-11-27

**Authors:** Yan Liu, Wenguang Zhang, Kai Wang, Anna Du

**Affiliations:** 1 Liaoning Provincial Key Laboratory of Advanced Material Preparation Technology, Shenyang University, Shenyang 110044, China; 2 School of Mechanical Engineering, Shenyang University, Shenyang 110044, China; 15942070723@163.com (W.Z.);

**Keywords:** inclusions, welding heat-affected zone, pressure vessel steel, intragranular acicular ferrite, nucleation mechanism

## Abstract

With the development of the pressure vessel industry, high-energy wire welding has a great future. However, this means higher demands on the weldability of pressure vessel steels. Controlling inclusions via oxidative metallurgy is a reliable method of improving the weldability of pressure vessel steels. Hence, in this paper, experimental steels with different Mg element mass fractions were prepared using vacuum metallurgy. Simulated welding for high-heat input welding was carried out using the Gleeble-2000 welding thermal simulation test machine. The inclusions in the welding heat-affected zone (HAZ) in the experimental steels were observed using an optical microscope (OM) and scanning electron microscope (SEM). The compositions of the inclusions were analyzed using an energy-dispersive spectrometer (EDS). The research results indicated that the addition of Mg could increase the number density of the inclusions in the welding HAZ. With the addition of Mg from 0 to 5 wt.%, the total number density of the inclusions increased from 133 to 687 pieces/mm^2,^ and the number density of the inclusions with a size of 0–5 μm^2^ increased from 122 to 579 pieces/mm^2^. The inclusions in the experimental steel welding HAZ with Mg elements were mainly elliptical composite inclusions composed of (Mg-Zr-O) + MnS. Moreover, MnS precipitated on the surface of the Mg-containing inclusions in the welding HAZ. Intragranular acicular ferrite (IAF) nucleation was primarily induced via the minimum lattice mismatch mechanism, supplemented with stress-strain energy and inert interface energy mechanisms.

## 1. Introduction

Pressure vessel steel is used to manufacture pressure vessels or other similar equipment, such as petroleum, gas storage, and transportation [1,2]. As production demands increase, traditional welding methods can no longer meet the requirements. In contrast, high-energy welding can significantly improve welding efficiency [3], reduce the number of passes, and shorten the welding cycle due to its advantage of higher heat input [4,5]. However, the higher wire energy and slower cooling rate lead to a weakening of the low-temperature impact properties of pressure vessel steels in the welding heat-affected zone. This has led to increasing demands on the weldability of metallic materials [6,7]. Therefore, it is necessary to develop a large wire energy welding pressure vessel steel [8,9].

To improve the weldability of pressure vessel steels and achieve the objective of increasing the toughness of the welding HAZ, the addition of particles to form dispersed and fine inclusions has been successful. As summarized in the literature [10,11,12], there are two main ways of achieving this objective: firstly, during welding thermal cycling, the pinning effect of fine inclusions dispersed into the base material is used to inhibit the growth of austenite grains, thereby refining the grains and improving the toughness of the welding HAZ. Secondly, in the post-weld cooling stage, in the austenite to ferrite phase transition process, some of the suitably sized inclusions promote the nucleation of IAF. The splitting effect of IAF subsequently causes the grain size to be refined. The IAF structure not only has a good toughness but also helps to improve the toughness of the pressure vessel steel welding HAZ.

To obtain the right type of inclusions to induce IAF, many researchers have controlled the formation of inclusions by adding deoxidizing elements, such as Ti, Zr, and Mg, to steel [13,14,15]. For instance, Qi et al. [16] investigated the effect of Ti treatment on inclusions in the laser-MAG hybrid welds of X100 pipeline steel. The results showed that with increasing Ti content, the composition of the outer inclusions evolved from mainly Al_2_O_3_ to Ti_2_O_3_ and finally to TiC. Acicular ferrite was induced to nucleate by Ti_2_O_3_ to form Mn-depleted zones, whereas Al_2_O_3_ and TiC could not nucleate via this mechanism. Yao et al. [17] investigated the effect of Zr addition on the inclusions of high-Ti low-alloy steel in a simulated HAZ. The main reason for the increase in toughness in the HAZ of coarse-grained Zr-containing steels is the formation of acicular ferrite-refined grains from 1–3 μm Al-Ti-Zr-O inclusions. However, the element Mg is more stable at high welding temperatures than the oxide of Ti. And compared to Ti and zirconium, elemental magnesium offers better economics and more effective resource conservation. Xu et al. reported the effect of MgO nanoparticles on the inclusions of the HAZ of shipplate steel [18]. The results showed that the addition of nanoparticles significantly optimized the structure of the inclusions, and the average size of inclusions was significantly reduced. The (Mg-Al-Ti)O inclusions of 1.2 μm size could effectively induce acicular ferrite and at the same time, improve the strength and toughness of HAZ. Lou et al. [19] researched the effect of a Ti-Mg-Ca treatment on the HAZ properties of C-Mn steels welded with high wire energy. It was found that the number density of the inclusions in Ti-Mg-Ca steel were significantly greater than those in Ti-Ca and C-Mn steels. In situ observations showed that MgO-containing oxide particles were more effective in inhibiting grain growth and providing a nucleation core in Ti-Mg-Ca steel than in Ti-Ca steel. However, there are very few studies on the effect of Mg treatment on the inclusions of steel HAZs for pressure vessels. And the mechanism of IAF nucleation induced by Mg-containing inclusions in pressure vessel steels is not yet fully understood and requires further theoretical analysis.

The formation of a microstructure in the welding HAZ of pressure vessel steels is affected by the type and size of the inclusions that serve as the core of IAF nucleation [20,21,22]. When welding a HAZ, IAF nucleation mainly involves four mechanisms: the minimum mismatch mechanism, the stress-strain energy mechanism, the local component change mechanism, and the inert surface mechanism. These four IAF nucleation mechanisms are intrinsically related to the presence of inclusions. Relying on only one mechanism often fails to fully elucidate the true nucleation process of IAF in the welding HAZ. Thus, it is unlikely that IAF nucleation occurs through a single mechanism, but it is certainly the result of a combination of these mechanisms.

In this paper, the effects of Mg elements on the inclusion characteristics (shape, composition, quantity, and size distribution) of the welding HAZs for pressure vessel steels were studied. The mechanisms of IAF nucleation induced by the inclusions containing Mg in the welding HAZ of pressure vessel steels were investigated, and a reference is provided for the design of the composition of pressure vessel steels for high-heat input welding.

## 2. Materials and Methods

**Figure 1:**
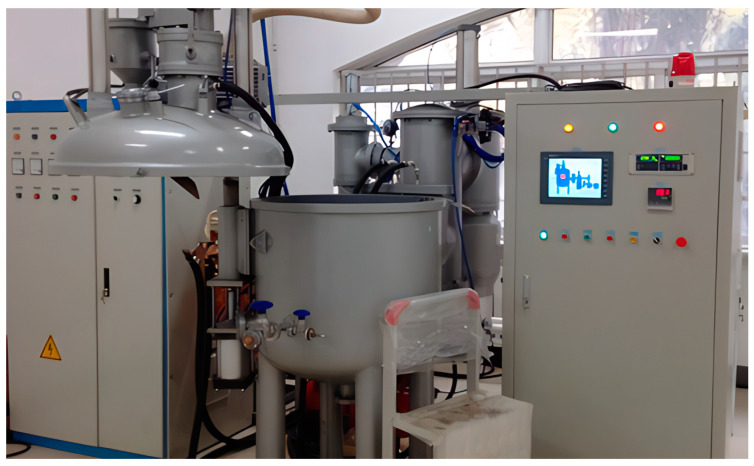
BJ-VIM-5 type vacuum induction melting furnace.

This experiment used the BJ-VIM-5 vacuum induction melting furnace (Dalunte Vaccum Technology, Shenyang, China) to smelt the experimental steel. The device is shown in Figure 1. The designed and actual smelting chemical compositions of the experimental steels are listed in Table 1.

In this experiment, the alkaline earth element Mg was added as the deoxidizer. According to the different mass fractions of Mg elements in the experimental steels, three groups of experimental steels were designed to be smelted, and each group smelted 5 kg. The designed addition amount (wt.%) of Mg for each group of experimental steels is shown in Table 2. The experimental steels without Mg addition, added 1 wt.% Mg, added 3 wt.% Mg, and added 5 wt.% Mg are called R, M1, M3, and M5, respectively.

**Figure 2:**
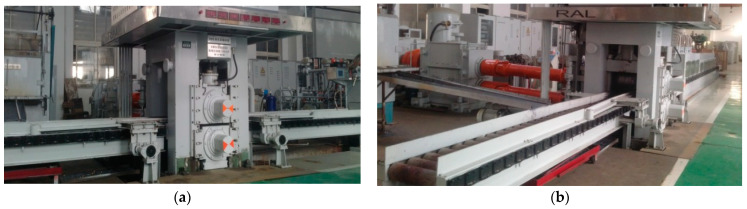
Controlled rolling and cooling equipment: (**a**) hot-rolling experimental unit with a diameter of 450 mm; (**b**) controlling cooling system of hot rolling.

The controlled rolling and cooling equipment (Northeastern University, Shenyang, China) are shown in Figure 2. The experimental steel billet was rolled on a hot-rolling test machine with a roll diameter of 450 mm. In the controlled rolling and cooling process, the billet was first heated to 900 °C in a heat treatment furnace and held for 35 min. After this, the furnace temperature was raised to 1200 °C and held for one hour. After seven controlled rolling passes, the billet was cooled to 380 °C via water cooling. Finally, the billets were cooled to room temperature in the air.

In order to obtain a sample of the designated area of the welding HAZ of sufficient size and to analyze it, the method of welding heat simulation is usually used [23,24]. It can simulate not only the process of a welding thermal cycle but also the stress and strain during welding according to the actual welding process. At the same time, it has the advantages of high accuracy, complete functions, and rapid warming and cooling.

**Figure 3:**
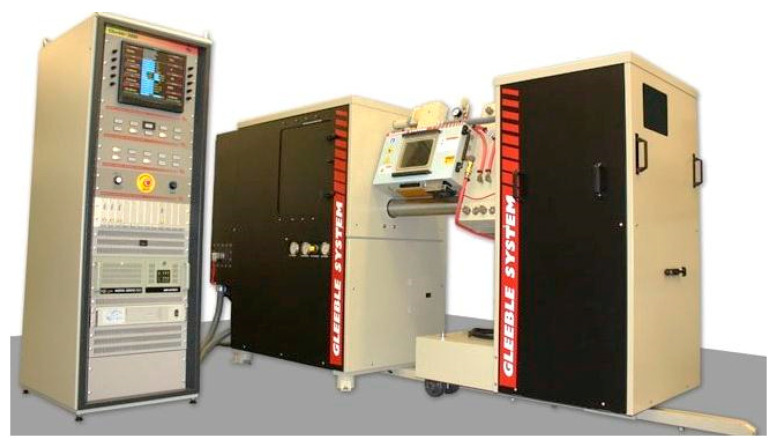
The Gleeble-2000 welding thermal simulation machine.

As shown in Figure 3, the Gleeble-2000 welding thermal simulation test machine(DSI, St. Paul, MN, USA) was used in this experiment. The machine utilizes resistance wire heating to achieve dynamic specimen thermal simulation through the effective control of the temperature. During the thermal simulation process of the experimental steels, firstly, the welding heat input was set to 100 kJ/cm, and the specimen was fixed. The second step was to preheat to 100 °C. The third step was to heat, with a heating speed of 200 °C/s. After 6 s, the peak temperature reached 1320 °C. The fourth step was to cool, and the cooling time t_8/5_ was set to 80 s. The fifth step was to gradually cool down from 500 °C to room temperature. 

**Figure 4:**
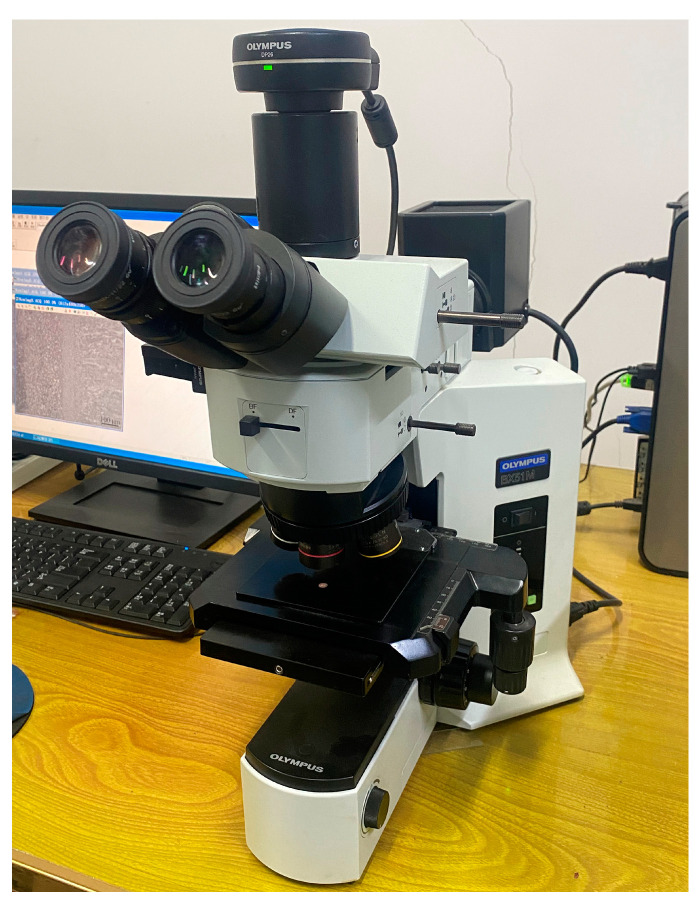
The OLYMPUS-CK40M optical microscope.

The inclusions in the welding HAZ were visualized using an OLYMPUS-CK40M optical microscope (Olympus, Tokyo, Japan), as shown in Figure 4. According to GB/T10561-2005 [25], the steel determination of the content of nonmetallic inclusions is achieved via the micrographic method of standard diagrams. IPP (Image-Pro Plus) 6.0 professional image analysis software was used to achieve a two-dimensional analysis of the quantity and size distribution of the inclusions in each experimental steel welding HAZ. It uses the ‘count and measure’ tool in IPP 6.0 software to measure the number and area of the inclusions and imports the measured data into an Excel table through the ‘data to clipboard’ action for the statistical analysis of the size distribution of the inclusions. The appearance and composition of the inclusions in welding HAZ of each experimental steel were observed and analyzed using an S-4800 SEM (Hitachi, Tokyo, Japan) and a supporting EDS (Hitachi, Tokyo, Japan). 

## 3. Results

### 3.1. Inclusions of the Experimental Steel in the Welding HAZ

The inclusions in the experimental steel welding HAZ were studied in terms of the number and size of the inclusions. Combining the results of related studies [26,27], the experiment specially adopted a method to measure the inclusion area, aiming for a more accurate reflection of the influence of inclusions on material welding HAZ properties while minimizing the inevitable statistical errors caused by inclusion shape factors. This was used as the basis for the size classification of the inclusions. The size distribution of the inclusions in the experimental steels is shown in Table 3.

The sum of the number density of the inclusions of each size is called the total number density of inclusions. As seen in Table 3, the number density of the inclusions in R is the lowest, only 133 pieces/mm^2^. The total number density of the inclusions in the welding HAZ of experimental steels M1, M3, and M5 with magnesium as the deoxidizer is higher than that in R, which is 291 pieces/mm^2^, 408 pieces/mm^2^, and 657 pieces/mm^2^, respectively. This shows that the addition of Mg elements promotes the formation of fine inclusions in the experimental steel welding HAZ to a certain extent under the present experimental conditions. Combining the results of related studies [28,29], it could be deduced that when the area of inclusions is less than 5 μm^2^, it is the most suitable size of inclusions to play the role of pinning grain boundaries and inducing IAF nucleation. These are called the effective inclusions.

**Figure 5:**
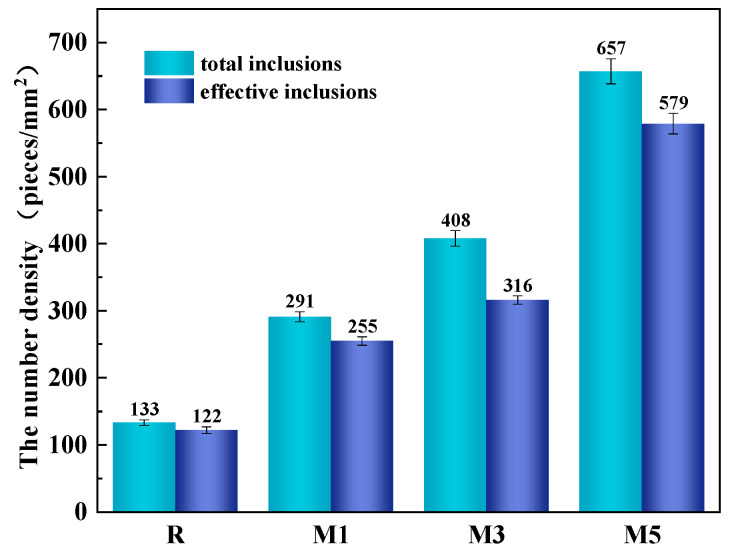
The number of total inclusions and effective inclusions per unit area in the experimental steel welding HAZ.

The changing trend of the number density of the total inclusions and the number density of effective inclusions of each experimental steel is shown in Figure 5. As shown in Figure 5, the number density of the effective inclusions is 122 pieces/mm^2^ for R. In addition, as the Mg content increases, the density number of the effective inclusions in the welding HAZ of the experimental steel increases: 255 pieces/mm^2^ for M1 and 316 pieces/mm^2^ for M2. When the mass fraction of 5wt.% Mg is added in the welding HAZ of the experimental steel, and the number density of the effective inclusions is also the largest, with 579 pieces/mm^2^. 

**Figure 6:**
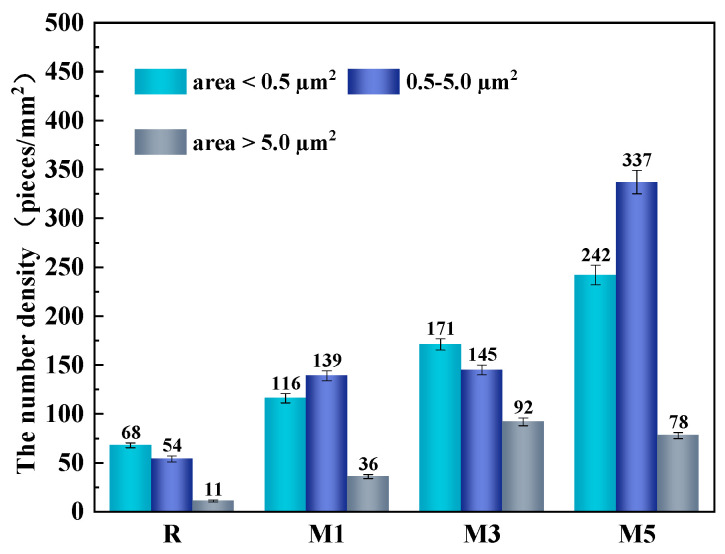
Distribution of inclusions with different effects per unit area in the HAZ of each experimental steel.

Only the number density of the total inclusions and effective inclusions is discussed above. To reflect more specifically the effect of inclusions on the strength and toughness of the welding HAZ in each experiment, the inclusions are categorized according to their different roles in different size ranges. When the area of the inclusion is less than 0.5 μm^2^, these nanoscale size inclusions could play the role of pinning grain boundaries and inhibit grain growth and coarsening in the welding HAZ. When the area of the inclusion is 0.5–5 μm^2^, these micro-grade inclusions are at the most suitable size for inducing IAF nucleation. The more IAF microstructures in the welding HAZ, the better the strength and toughness. When the area of the inclusion is greater than 5 μm^2^, these larger-sized inclusions are not very effective in pinning grain boundaries and inducing IAF nucleation [30]. Therefore, the number density of inclusions of this size in the experimental steel welding HAZ should be minimized. The distribution of inclusions with different effects per unit area in the HAZ of each experimental steel is shown in Figure 6.

As shown in Figure 6, the total number density of inclusions is the least in the R steel welding HAZ. The number density of inclusions is 68 pieces/mm^2^ for areas less than 0.5 μm^2^ and 54 pieces/mm^2^ for areas in the 0.5–5 μm^2^ range. Therefore, the number density of inclusions that both pin and induce IAF nucleation is relatively minimal, which leads to the poor strength and toughness of the R steel. In contrast, in the three experimental steels with Mg elements, the number of inclusions that perform the role of pinning and inducing IAF nucleation increases gradually, and both are significantly higher than the R steel, except that the number of pinning inclusions in the welding HAZ of the experimental steel with 3 wt.% Mg is higher than that of the inclusions which induce IAF nucleation. The inclusions in the remaining two groups of experimental steels with Mg elements are mainly used for inducing IAF nucleation. Among them, the 116 pieces/mm^2^, 171 pieces/mm^2^, and 242 pieces/mm^2^ inclusions with an area less than 0.5 μm^2^ were found in the M1, M3, and M5 steels, respectively. The number density of the inclusions in the 0.5–5 μm^2^ interval was 139 pieces/mm^2^, 145 pieces/mm^2^, and 337 pieces/mm^2^ for M1, M3, and M5, respectively. It was observed that the addition of Mg elements facilitates the production of more inclusions that induce IAF nucleation in the experimental steel welding HAZ.

### 3.2. EDS Analysis of the Main Inclusion Composition

**Figure 7:**
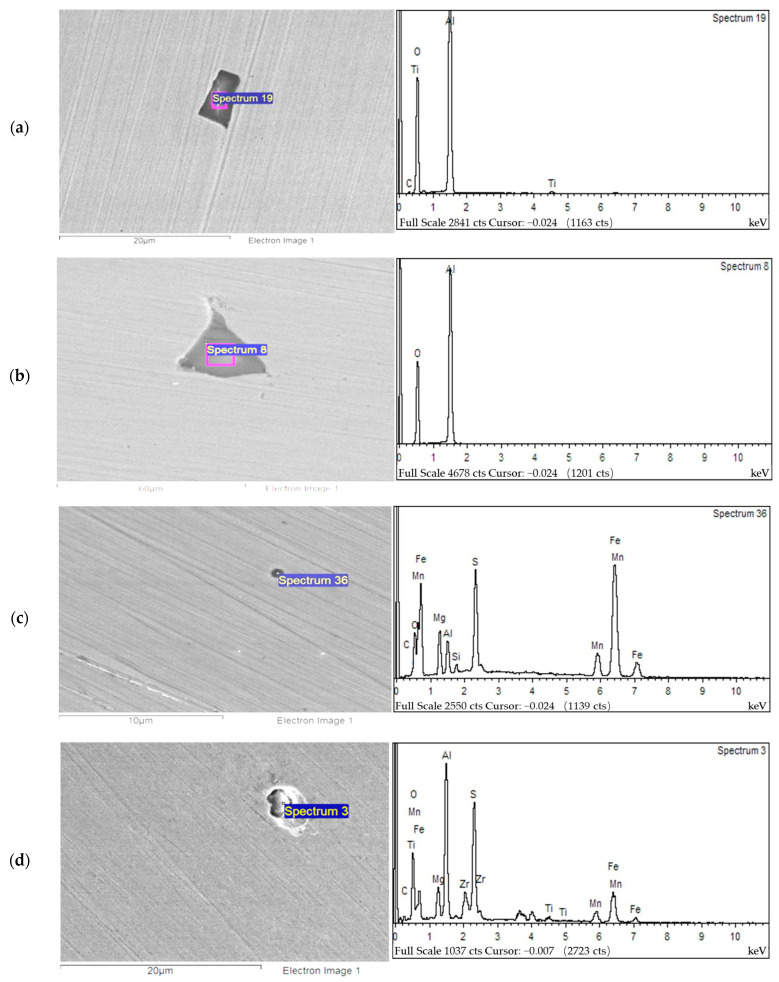
The main inclusion composition of the HAZ in each experimental steel: (**a**,**b**) are R steel, while (**c**,**d**) are steels with added Mg.

In this experiment, an S-4800 SEM and a supporting EDS were used to research the R steel following emery paper grinding and mechanical polishing, as well as the specimen of the welding HAZ in the experimental steel containing Mg. The main inclusions were found for the shape observation and composition analysis. The results are shown in Figure 7.

As shown in Figure 7a,b, the main inclusion in the welding HAZ of R steel is the oxide inclusion of Ti or Al elements, and it has a very irregular shape, large size, and irregular quadrangles with edges or even triangular angles. The oxide of Al (Al_2_O_3_) is prone to accumulate in steel fluid to form coarse inclusions. This inclusion has no induction effect on IAF, and it is easy to form cracks during processing, which damages the toughness of the welding HAZ of the material to a certain extent and is an undesirable inclusion. As shown in Figure 7c,d, the inclusions in the experimental steel welding HAZ with the Mg elements added as a deoxidant are mainly MgO or composite inclusions of Mg, Zr, and Al. There are also some MnS inclusions. Mg belongs to an alkaline earth metal element. Combined with Figure 7c, the shape of the MgO inclusion is generally spherical or elliptical. It favors the IAF nucleation of a ductile microstructure in the welding HAZ. Therefore, the addition of an Mg element could improve the toughness of the welding HAZ of experimental steels. 

The alkaline earth metal Mg causes the shape of the inclusion to be mostly spherical, with a very small size of approximately 1.5 μm, which is in the most suitable size range of inclusions for IAF nucleation as described above. Moreover, there are more inclusions of this size in the experimental steel welding HAZ, and the amount of IAF induced, therefore, is also large, which is conducive to the improvement of the strength and toughness of the experimental steel welding HAZ.

## 4. Discussion

### 4.1. The Effect of Adding Mg on the Welding HAZ of Experimental Steels

**Figure 8:**
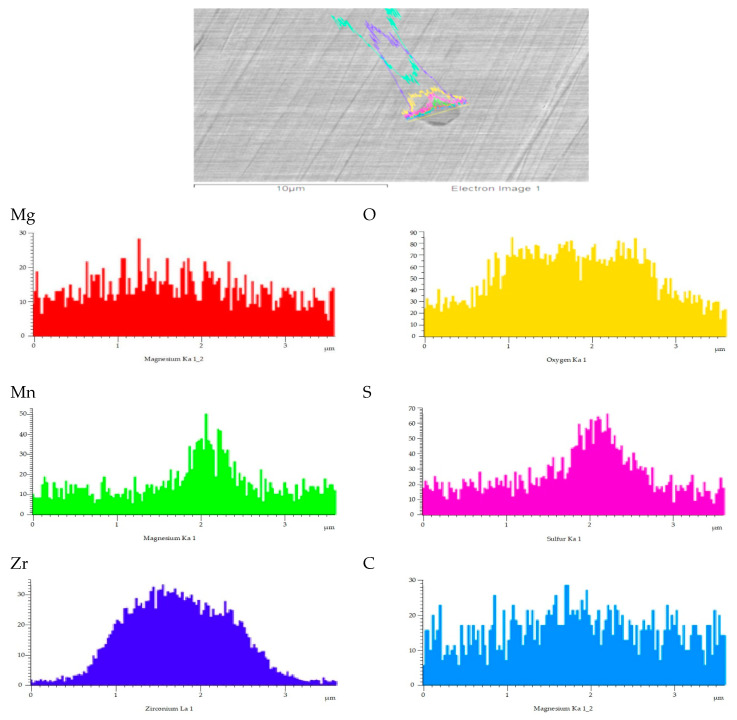
Typical inclusion morphology and line scanning results of the Mg-added steel.

The morphology and line scanning analysis of typical inclusions in the welding HAZ of experimental steels with Mg are shown in Figure 8. The X-axis of Figure 8 represents the line scanning distance centered on the inclusions. It was observed that the content of Mg elements is the highest in the central area of the inclusion line scanning, and the content gradually decreases from the center to the sides. Both the Zr and O elemental content show an apparent increasing trend in the center region. The variation trend of the Mn and S elements is roughly the same, and the positions of higher-content Mn and S elements are slightly behind the central area of the Mg, Zr, and O elements. Therefore, the basic morphology of the inclusions could be identified as a compound inclusion mainly containing (Mg-Zr-O) formed in the central position, with MnS inclusions precipitating to the right of the center of the inclusions. Under the condition of line scanning, the typical inclusions in the welding HAZ of the experimental steels with Mg elements are elliptical (Mg-Zr-O) + MnS compound inclusions. The Mg and their compound inclusions in the welding HAZ mainly play the role of fine-grain strengthening and dispersion strengthening.

By observing the morphology and line scanning results of the typical inclusions in the welding HAZ of the experimental steel with Mg added, the following results were found: firstly, from the morphology of the inclusions, spherical or nearly spherical inclusions are produced. The reason is that the spheroidization of Mg elements as alkaline earth metals changes the morphology of the inclusions in the welding HAZ of all experimental steels. Secondly, from the line scanning distribution of the elements in the inclusions, Mg elements are generally combined with other elements to form (Mg-Zr-O) + MnS composite inclusions. 

### 4.2. The Mechanism of IAF Nucleation Induced by Mg Elements in the Welding HAZ

**Figure 9:**
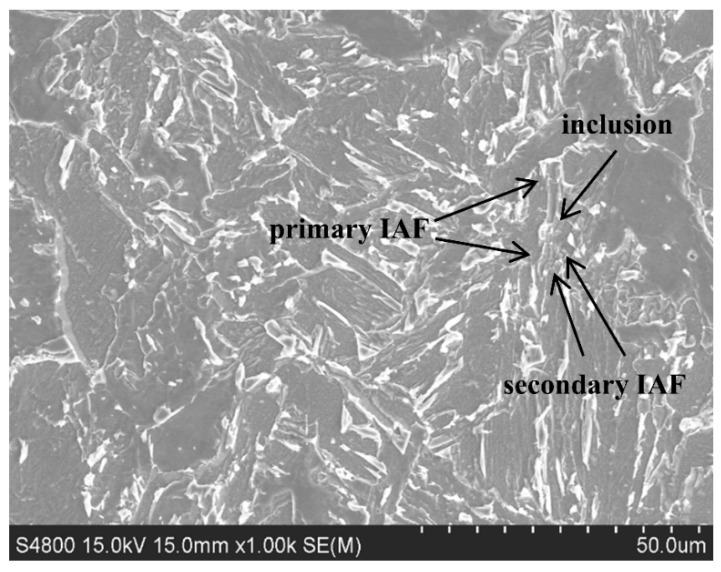
Typical microstructure of IAF in the experimental steel welding HAZ.

After corroding the welding HAZ metallographic specimen of the experimental steels with Mg added to them, the typical IAF microstructure formed by the fine circular inclusions was found, as shown in Figure 9.

The addition of Mg could promote the spheroidization of the inclusions. The (Mg-Zr-O) composite inclusions containing Mg could effectively induce IAF nucleation and improve the strength and toughness of the welding HAZ in experimental steels. It can be clearly seen from the position of the inclusion that two more ‘bulky’ primary IAF microstructures are formed. Secondary IAF microstructures are formed via the inductive nucleation mode of primary IAF microstructures. The relatively ‘thick’ primary IAF microstructure is the main trunk, and, at the same time, many branch IAF microstructures continue to be induced on the trunk. This is the refinement effect of the IAF microstructure itself. Many IAF microstructures are intertwined and firmly combined with each other, playing a role in improving the strength and toughness of the HAZ in experimental steel welding. 

#### 4.2.1. IAF Nucleation Mechanism Induced via minimum Lattice Mismatch

The inclusions that promote IAF nucleation are affected by the interface energy of themselves or between the precipitates and the ferrite, and the interface energy between them is determined via lattice mismatch. It is shown that [31,32] the inclusions with a good coherent relationship with the IAF microstructure or the precipitates on them could effectively reduce the interface energy between the inclusions and ferrite and promote IAF nucleation. The lattice mismatch of the inclusions and ferrite in experimental steels is shown in Table 4.

As shown in Table 4, there is a good coherent relationship between the inclusions of some body-centered cubic microstructures, such as MnS and ferrite. The lattice mismatch degree is small, which is beneficial for becoming the core of IAF nucleation and promoting the formation of IAF microstructures in the welding HAZ. In this experiment, the lattice mismatch between MgO inclusions and ferrite is only 2.8%, which is even smaller than that of MnS. Furthermore, through the line scanning analysis of the composite inclusions containing Mg elements in the welding HAZ, it was found that MnS inclusions are precipitated from the surface of Mg-containing composite inclusions, which reduces the mismatch between the composite inclusions and ferrite and promotes the formation of the IAF microstructure in the welding HAZ. Therefore, the minimum mismatch mechanism is suitable for the induction of IAF nucleation in the welding HAZ of Mg-containing steels.

#### 4.2.2. IAF Nucleation Mechanism Induced via Stress-Strain Energy

Due to the high density of dislocations in the IAF microstructure, the average coefficient of the thermal expansion of the inclusions during high-heat input welding is small, whereas the coefficient of thermal expansion of the austenitic matrix is relatively large. Thus, a stress-strain field is formed around the inclusion, causing a certain degree of distortion. The resulting distortion could provide activation energy to induce IAF nucleation. Data [36,37] shows that, as shown in Table 5, the average thermal expansion coefficient of austenite is 23.0 × 10^−6^ K^−1^, while that of the MgO is 13.8 × 10^−6^ K^−1^, which is only close to half of that of austenite. Thus, a large stress-strain field is generated around the inclusion to power IAF nucleation. However, it is also taken into account that the energy required for ferrite nucleation is often one to two orders of magnitude larger than the activation energy generated by thermal expansion differences alone. Therefore, stress-strain energy only exists as an auxiliary mechanism to induce IAF nucleation in the welding HAZ of experimental steels with Mg elements.

#### 4.2.3. IAF Nucleation Mechanism Induced via Local Compositional Change

**Figure 10:**
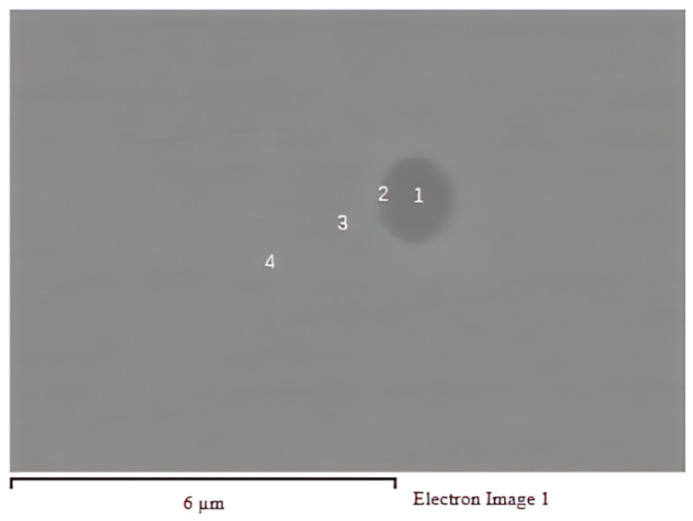
Position map of the inclusion point analysis of the welding HAZ in Mg-added steels.

Mn elements are poor around inclusions. The Mn content is significantly reduced within a certain range, and a poor Mn area is formed. This would produce local composition changes and induce IAF nucleation [38,39]. Therefore, the inclusions in the experimental steel with the addition of the Mg element were analyzed using EDS composition point analysis. The position map of the inclusion points analysis of the welding HAZ in added Mg steels is shown in Figure 10. Point 1 is at the center of the circular inclusions, point 2 is at the edge of the inclusions, point 3 is closer to the outside of the inclusions, and point 4 is at the matrix. The inclusion of chemical composition point analysis results of the welding HAZ in the Mg-added steels are shown in Table 6.

As shown in Table 6, the content of the Mn element at point 1 is lower than the Mn content at point 4, indicating that the Mn element is not enriched on the inclusions. The Mn content at point 2 is equivalent to that of the matrix. The Mn elemental content at point 3 is slightly higher than the Mn content of the matrix, but the change is not obvious. Therefore, it could be concluded that there is no Mn-poor area around the inclusions containing Mg, and this theory cannot be used as a mechanism to explain the induction of IAF nucleation in the welding HAZ of Mg-containing steels.

#### 4.2.4. IAF Nucleation Mechanism Induced via Inert Interface Energy

Ricks et al. [40] suggested that the inclusion of a surface in steel as an inert interface provides energy to induce IAF nucleation. The interface between the inclusions and the matrix plays a role in reducing the nucleation barrier of IAF, and it is only related to the number and size of the inclusions, not their composition. Large amounts of small spherical inclusions formed by adding Mg elements are distributed dispersedly in the welding HAZ. It could partly provide a high-energy inert surface for IAF nucleation to some extent. It could reduce the IAF nucleation barrier and promote IAF nucleation. According to the relevant literature [40,41], the inert interface energy of complex inclusions with diameters of 0.5–2.0 μm is approximately 10^5^ to 10^6^ J/mol. This value is close to the lowest energy required for inducing IAF nucleation (approximately 10^6^ J/mol). However, simply relying on an inert interface energy mechanism is not enough to fully explain the nucleation mechanism of IAF microstructures.

## 5. Conclusions

(1) With increasing Mg content, the number density of the total inclusions and the number density of the effective inclusions in the experimental steel welding HAZ show an upward trend. The total number density of the inclusions and the number of effective density inclusions in the welding HAZ of the experimental steel without added Mg are the lowest, with 132 pieces/mm^2^ and 122 pieces/mm^2^, respectively. In contrast, the welding HAZ of the experimental steel with 5 wt.% Mg added exhibits the highest total number density of inclusions and the number density of effective inclusions, amounting to 656.39 pieces/mm^2^ and 579.10 pieces/mm^2^, respectively.
(2) In the three experimental steels with Mg elements, the number density of the inclusions that play the role of pinning and inducing IAF nucleation increases gradually and is markedly higher than that of the R steel. The addition of Mg elements is beneficial to produce more inclusions inducing IAF nucleation in the welding HAZ of the experimental steel under the experimental conditions.
(3) The inclusions in the experimental steel welding HAZ with Mg elements are mainly elliptical composite inclusions composed of (Mg-Zr-O) + MnS with a very small size of approximately 1.5 μm. The inclusions with such shape and size are conducive to the nucleation and growth of ductile IAF microstructures.
(4) After the addition of Mg elements, MnS is precipitated on the surface of Mg-containing inclusions in the welding HAZ. IAF nucleation is induced mainly via a minimum lattice mismatch mechanism, supplemented by stress-strain energy and inert interface energy mechanisms.

## Figures and Tables

**Table 1:** Actual melting chemical compositions of experimental steels.

Items	Chemical Compositions (wt.%)												
	C	Si	Mn	P	S	Ni	Mo	V	Nb	Cr	Als	Ti	N
Design compositions	0.06–0.09	0.10–0.20	1.45–1.60	≤0.015	≤0.006	0.25–0.35	0.20–0.30	0.045–0.055	0.01–0.02	0.10–0.30	≤0.01	0.01–0.02	≤0.005
Actual compositions	0.10	0.13	1.4	0.012	0.005	0.28	0.202	0.047	0.015	0.19	0.007	0.016	/

**Table 2:** Designed magnesium content of the experimental steels.

Items	R	M1	M3	M5
Mg (wt.%)	0	1	3	5
Mg-Zr alloys (g)	0	71.754	215.261	358.770

**Table 3:** Size distribution of HAZ inclusions in each experimental steel.

	Items	R	M1	M3	M5
Size (μm^2^)					
<0.5		68	116	171	242
0.5–1.0		26	77	76	137
1.0–2.0		12	44	33	82
2.0–3.0		9	8	13	60
3.0–5.0		7	10	23	58
5.0–10.0		7	9	23	36
10.0–20.0		2	22	37	24
>20		2	5	32	18

**Table 4:** Lattice mismatch of the inclusions and ferrite in experimental steels [33,34,35].

Inclusion	Lattice Constant/(10^−8^ cm)			Mismatch (%)
	a	b	c	
MnS	5.224	5.224	5.224	8.8
MgO	4.216	4.216	4.216	2.8

**Table 5:** Contrast of the average thermal expansion coefficients.

Substance	Austenite	MgO
Thermal expansion coefficient (10^−6^ K^−1^)	23.2	13.8

**Table 6:** Inclusion of chemical composition point analysis results of the welding HAZ in Mg-added steels.

Position	S	Mn	Mg	Fe
1	0.66	0.59	2.66	39.34
2	0.80	0.71	2.23	60.37
3	0.69	0.82	0.56	79.69
4	-	0.75	-	98.34

## Data Availability

Data are included in the article.
